# Gastrointestinal tract organoids as novel tools in drug discovery

**DOI:** 10.3389/fphar.2024.1463114

**Published:** 2024-08-30

**Authors:** Li Zhou, Dan Luo, Wei Lu, Jun Han, Maoyuan Zhao, Xueyi Li, Tao Shen, Zhao Jin, Jinhao Zeng, Yueqiang Wen

**Affiliations:** ^1^ School of Basic Medicine, Chengdu University of Traditional Chinese Medicine, Chengdu, China; ^2^ Department of Elderly Care Center, Chengdu Pidu District Hospital of Traditional Chinese Medicine, Chengdu, China; ^3^ Department of Geriatrics, Hospital of Chengdu University of Traditional Chinese Medicine, Chengdu, China; ^4^ Department of Pediatrics, Guang’an Hospital of Traditional Chinese Medicine, Guang’an, China

**Keywords:** organoids, gastrointestinal, drug discovery, disease modeling, precision medicine

## Abstract

Organoids, characterized by their high physiological attributes, effectively preserve the genetic characteristics, physiological structure, and function of the simulated organs. Since the inception of small intestine organoids, other organoids for organs including the liver, lungs, stomach, and pancreas have subsequently been developed. However, a comprehensive summary and discussion of research findings on gastrointestinal tract (GIT) organoids as disease models and drug screening platforms is currently lacking. Herein, in this review, we address diseases related to GIT organoid simulation and highlight the notable advancements that have been made in drug screening and pharmacokinetics, as well as in disease research and treatment using GIT organoids. Organoids of GIT diseases, including inflammatory bowel disease, irritable bowel syndrome, necrotizing enterocolitis, and *Helicobacter pylori* infection, have been successfully constructed. These models have facilitated the study of the mechanisms and effects of various drugs, such as metformin, Schisandrin C, and prednisolone, in these diseases. Furthermore, GIT organoids have been used to investigate viruses that elicit GIT reactions, including Norovirus, SARS-CoV-2, and rotavirus. Previous studies by using GIT organoids have shown that dasabuvir, gemcitabine, and imatinib possess the capability to inhibit viral replication. Notably, GIT organoids can mimic GIT responses to therapeutic drugs at the onset of disease. The GIT toxicities of compounds like gefitinib, doxorubicin, and sunset yellow have also been evaluated. Additionally, these organoids are instrumental for the study of immune regulation, post-radiation intestinal epithelial repair, treatment for cystic fibrosis and diabetes, the development of novel drug delivery systems, and research into the GIT microbiome. The recent use of conditioned media as a culture method for replacing recombinant hepatocyte growth factor has significantly reduced the cost associated with human GIT organoid culture. This advancement paves the way for large-scale culture and compound screening of GIT organoids. Despite the ongoing challenges in GIT organoid development (e.g., their inability to exist in pairs, limited cell types, and singular drug exposure mode), these organoids hold considerable potential for drug screening. The use of GIT organoids in this context holds great promises to enhance the precision of medical treatments for patients living with GIT diseases.

## 1 Introduction

Organoids are organ-specific cell cultures developed from *in vitro* pluripotent stem cells (PSCs) or pluripotent adult stem cells (ASCs) to mimic the structure and function of their corresponding *in vivo* organ (Sato et al., 2009). These cells are cultured in specific *in vitro* environments to form tiny cell populations that self-organize and differentiate into functional cell types (Schutgens and Clevers, 2020). These organoids exhibit highly physiological properties that recapitulate the differentiation capacity of cells, tissue structures, as well as the interactions between cells and between cells and matrices (Günther et al., 2022). Gastrointestinal tract (GIT) organoids were first developed in Hans Clever’s laboratory (Yi et al., 2021), where researchers successfully constructed intestinal organoids with intestinal crypt-villus structures *in vitro* by extracting Lgr5+ ASCs directly from the intestines and culturing them with appropriate growth factors and supportive substrates (Sato et al., 2009). Since the development of intestinal organoids, various types of organoid models, such as those for the esophagus, lung, liver, stomach, pancreas, and colorectum, have also been developed (Clevers, 2016). These models provide more accurate biological representations to aid research in areas such as immunotherapy, new drug discovery, and drug screening (Tang et al., 2022).

GIT organoids have advantages over two-dimensional (2D) cell systems and intestinal explant models. The primary drawbacks of 2D cell systems is that they lack many of the characteristics of normal GIT epithelium, they contain a single cell type, they lack the complex structure of *in vivo* tissues (Sato et al., 2009; Rossi et al., 2018), and they have a different genetic profile than normal cells (Millen et al., 2023; Sommerkamp et al., 2021). In contrast, intestinal exosomes reflect the complex structure of the *in vivo* intestinal tract, but they do not support passaging cultures (Sato et al., 2009; Xi et al., 2021). The development of GIT organoids remedies these deficiencies, and the model has numerous proven advantages (Sato et al., 2009). GIT organoids exhibit high similarity to the original tissue and can represent the patient cohort, capturing diversity. The high similarity between GIT organoids and the original tissue is reflected in aspects such as anatomical morphology, cellular composition, physiological function, and gene expression patterns. GIT organoids contain most intestinal epithelial cell types, including absorptive cells, cup cells, pan cells, and tufted cells, and have crypt-like structures and villous regions that are key in *in vivo* human intestinal tissue (Filippello et al., 2022; Kim et al., 2022). GIT organoid models can also be cultured for more than 1.5 years during which time they remain functional (e.g., the motility, absorption, and secretion functions of the gastrointestinal tract.) (Elbadawi et al., 2021). The gene expression pattern of the GIT organoid is more similar to that observed in normal tissues than in 2D cell system or animal models (Oberdoerffer et al., 2008; Legnini et al., 2023). This model provides advantages for studying genomic and epigenomic host-environment interactions (Witonsky et al., 2023). GIT organoids represent the patient cohort, capture diversity, and are manifested in the following aspects: First, GIT organoids represent the personalized characteristics of the patient’s disease. GIT organoids derived from patient-specific induced pluripotent stem cells or directly from patient biopsy tissue enable them to represent the individual’s genetic background, potentially capture the patient-specific disease characteristics, and are excellent models for studying intestinal epithelial interactions (Deleu et al., 2023). Second, GIT organoids capture the diversity of diseases. By creating organoids from patients with different diseases, researchers can study a range of pathologies and pathological processes, including inflammatory bowel disease, gastrointestinal cancer, *H. pylori* (*Helicobacter pylori*) infection, and viral infections. GIT organoids also allow patient-derived viruses to exist and replicate efficiently, circumventing the limitation that the use of laboratory-adapted strains is not representative of all circulating strains (Yin et al., 2015). Ultimately, GIT organoids also capture the diversity of populations. Biobanks of organoids from different populations have been established (Yao et al., 2020), ensuring that research is representative of a broad population and understanding how different genetic backgrounds affect disease susceptibility and treatment responses. Due to the high accuracy of GIT organoid disease modeling (Belair et al., 2020), it has also been used to predict patient responses to therapy (Aalbers et al., 2022) and subsequent clinical outcomes, thereby providing therapeutic guidance (Moussa et al., 2020).

GIT organoids have been increasingly used in disease research and drug screening, but there is a lack of literature that summarizes and discusses data around the use of GIT organoids as disease models and drug screening platforms. Herein, we focused on GIT organoid modeling of related diseases, and found that a variety studies have successfully been carried out using organoid-based GIT disease models. Examples include, pharmacological studies into clinical treatments, with some helping to elucidate the pathogenic mechanisms of GIT diseases and screening for preventive and therapeutic drugs. Further, previous studies using such organoid models have helped to reveal the pathology of viral infections and the subsequent GIT responses, leading to the development of novel drugs that inhibit viral replication. These models have also been key for testing the GIT toxicity of drugs and guiding their use in clinical practice. Owing to their use in wide range of applications and the richness of the data they can yield, GIT organoid models are important tools in disease research, drug screening, pharmacokinetics, drug toxicology research, and clinical practice, among others, and promotes the development of precision medicine to improve global health.

## 2 Advances in organoid technology

**FIGURE 1:**
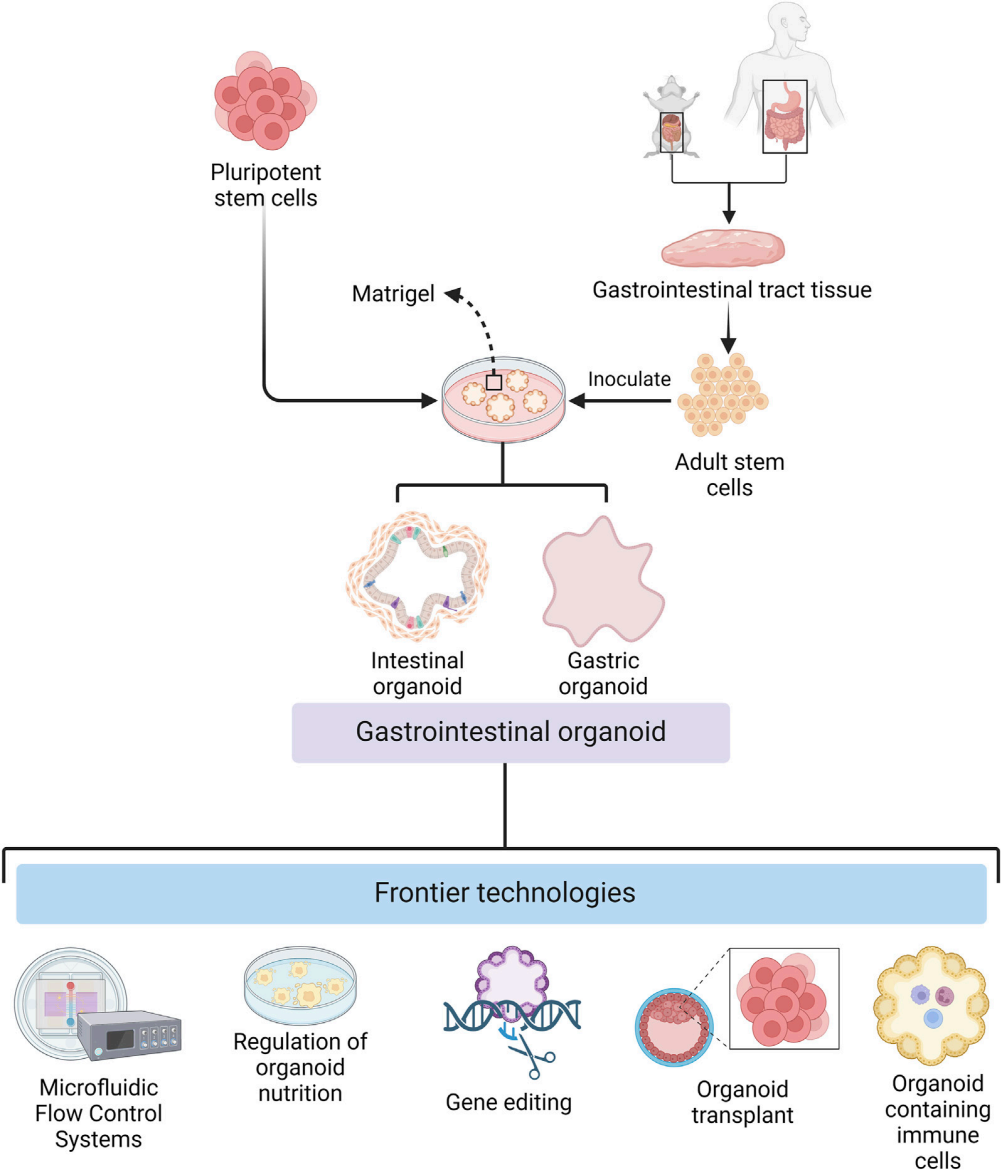
Cultivation of gastrointestinal organoids and frontier technologies (Created with BioRender.com).

There have been considerable developments in different aspects of organoid technology for basic research and clinical applications (Figure 1). For example, there have been breakthroughs in organoid culture technology, both in terms of broad applicability and specificity. In biomedicine, they have been transformed from ordinary *in vitro* models that can only be used for disease research to all-purpose models that can be used to study the mechanisms of viral and parasitic infections. The development of organoids that contain components of the immune system has further improved their utility as models for disease relevant and physiological *in vitro* research.

The development of organoid culture technology has promoted the wide application of organoids in basic research and clinical therapy. During organoid culture, cells can be attached to scaffolds composed of natural extracellular matrix or synthetic materials (Murakami and Masui, 1980; Giandomenico et al., 2019; Jaganathan et al., 2014; Garreta et al., 2021), or they can be aggregated to form microtissue spheroids by droplets, magnetic fields, or special synthetic materials. The use of collagen gels instead of matrix gels also makes low-cost mass replicable organoid models a reality (Takahashi et al., 2023), facilitates large-scale culture and complex screening of GIT organoids, and expands the application of GIT organoids in various research fields. The reduction in culture costs has been accompanied by an increase in the precision of culture conditions. Wuputra et al. (2021) developed precise culture conditions for gastric organoids, which have improved the accuracy of GIT organoids as models for clinical therapeutic and medical applications.

In addition to the development of culture techniques suitable for general GIT organoids, modifying the culture conditions to incorporate disease-specific features enhances their suitability for disease research applications. Certain disease studies necessitate specific environmental conditions, such as controlled oxygen concentrations, which require techniques to regulate the oxygen environment of the organoids. Li et al. (2014) innovatively used an air–liquid interface culture method for modeling colorectal cancer (CRC) organoids, which improves *in vitro* oxygenation and enables studies of hypoxic diseases with control of this variable. Also, Zheng et al. (2021) designed a microfluidic chip capable of precisely regulating oxygen concentrations in each chamber. These studies provide technical support for organoid as a platform for researching hypoxic diseases. Moreover, certain diseases necessitate specific nutritional environments. Perlman et al. (2023) developed a new technique for culturing malnourished gastric organoids, which can alter the nutritional status of the organoids and provide a basis for the use of organoids as a research tool for studying the effects of nutritional status on the GIT epithelium.

While the application of organoids in disease research is expanding, its use as a therapeutic tool for clinical diseases is also becoming more widespread. Huang et al. (2023) differentiated cultured human gastric stem cells into islet-like organoids for the treatment of diabetes mellitus. Moussa et al. (2020) utilized organoid transplants to repair intestinal post-radiation injuries, which provided the basis for the development of organoid regenerative medicine. In addition, GIT system-on-a-chip organoids have emerged as promising *in vitro* models for preclinical studies. These advances are based on recent developments in several technologies such as bioprinting, microfluidics and organoid research.

Organoid technology is continually evolving for general disease research, but researchers are also developing new experimental models, combining organoids with gene editing techniques, and facilitating the study of virus and parasitic infection mechanisms. Kim et al. (2022) developed bovine gastric organoids as a novel *in vitro* model to study host-parasite interactions in GIT nematode infections. Gebert et al. (2024) utilized a gene-encoded calcium indicator for real-time calcium imaging of virus-infected organoids to establish an adaptable platform to represent cellular signals in virus-infected GIT nematodes. Moreover, an encoded calcium indicator has also been used for real-time calcium imaging of virus-infected organoids, establishing a tractable method for characterizing cellular signals in virus-infected GIT organoids.

Organoid modeling has great potential in biomedicine; however the lack of a model immune system in these models is a major drawback (Günther et al., 2022). However, by transplanting PSC-derived human intestinal organoids (HIOs) under the kidney capsule of mice with a humanized immune system, Bouffi et al. found that human immune cells temporarily migrate to the mucosa and form cell aggregates similar to human intestinal lymphoid follicles. In addition, upon exposure to microorganisms, the number of epithelial microfollicular cells in this study increased, leading to immune cell activation and secretion of immunoglobulin A antibodies in the lumen of the HIOs. This immune cell-containing HIO system provides a framework for future studies of infection- or allergen-driven intestinal diseases (Bouffi et al., 2023), compensating for the lack of an immune system in organoids. Taken together, the continued advances in organoid technology are aided by the available comprehensive supporting methods, making it a valuable tool for biomedical research.

## 3 Models for GIT disease

### 3.1 GIT organoids as tools in inflammatory bowel disease (IBD) research

**FIGURE 2:**
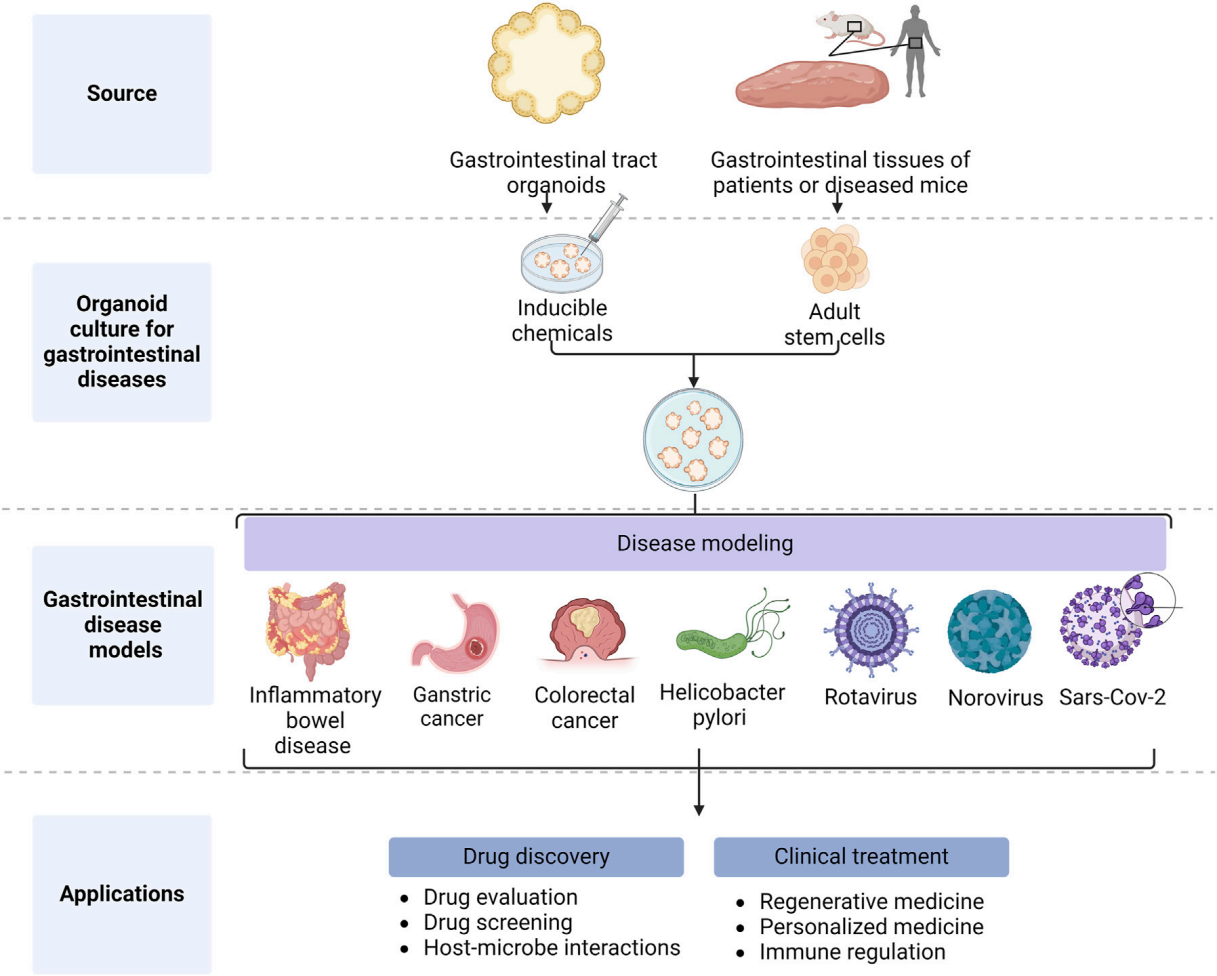
Application of gastrointestinal organoids (Created with BioRender.com).

GIT organoids are excellent models which have been used to study a variety of GIT diseases (Figure 2), including IBD (Holmberg et al., 2017) [i.e., Crohn’s disease (CD) and ulcerative colitis (UC)]. IBD manifests itself as recurrent episodes of inflammation and remission, all characterized by chronic inflammation in different parts of the GIT tract. These inflammatory episodes can cause diarrhea, abdominal pain, blood in the stool, and other symptoms. Although IBD pathogenesis remains unclear, previous studies have shown that immune dysfunction in the intestinal mucosa due to genetic and immunological factors plays an important role in IBD pathology (Iacucci et al., 2024).

The influence of genetic factors on IBD pathogenesis has been demonstrated by twin, targeted sequencing, and genome-wide association studies (GWAS) (Mokry et al., 2014). In 2013, an organoid-based genetic study of IBD was conducted by researchers who generated acetylated histone 3 lysine 27 profiles from primary intestinal epithelial cells and subsequently cultured organoid derived from these cells. From this, 92 out of 163 IBD-associated single-nucleotide polymorphisms (SNPs) were shown to be associated with differentially active regulatory elements. Moreover, variations in these SNPs were shown to create or disrupt known binding motifs, suggesting that they may affect the binding of transcriptional regulators, thereby altering the expression of regulated genes. In addition to variants in protein-coding genes, variants in noncoding DNA regulatory regions active in intestinal epithelial cells and immune cells may also be involved in IBD pathology (Mokry et al., 2014). With the wide study of GWAS’ and SNPs, many genes involved in immune regulation have been successfully identified as susceptibility genes for IBD, including Interleukin 28, which controls intestinal epithelial cell proliferation in mice with colitis and accelerates mucosal healing by activating the signal transducer and activator of transcription 1 protein (Chiriac et al., 2017).

Patients with IBD suffer from an imbalance in immune regulation, and the marked differences in the immune microenvironment between the two models of IBD (i.e., UC *versus* CD), are also reflected in T cell differentiation patterns (Biton et al., 2018). In organoid models of UC and CD, T cells either differentiate more into the Th17 or Th1 type, respectively. Interestingly, Hammoudi et al. demonstrated for the first time that autologous mucosal T cells can directly induce epithelial cell death in patients with CD (Hammoudi et al., 2022).

In conclusion, GIT organoids are important *in vitro* models for IBD research, where they are used to accurately simulate the effects of reductive genetic and immune factors on intestinal epithelial damage in IBD.

### 3.2 GIT organoids as tools in GIT cancer research

GIT tumor organoids provide an ideal *in vitro* model for studying GIT tumor cells and molecular signaling pathways. These organoids are able to maintain the complexity of GIT tumor cells and can recapitulate tumor biology.

Many studies have been carried out using organoids to mimic gastric cancer progression. Further, the combination of GIT organoids and gene editing technology has facilitated basic research into gastric cancer. Tong et al. induced normal gastric organoids to develop the malignant phenotype of gastric cancer by knocking down the tumor suppressor, *CDH1*, and they subsequently showed that knocking down *RHOA* restored them to their normal morphology (Tong et al., 2023). These data suggested that normal gastric organoids can develop into gastric cancer organoids, and their malignant behavior can be reverted to normal with relevant interventions, highlighting the importance of organoids in studying tumorigenesis and development. In patients with GIT tumors, gastric cancer peritoneal metastasis (GCPM) is a leading cause of death. During this process, monocyte-like dendritic cells (DCs) with pro-angiogenic effects are increased and their antigen-presenting capacity is reduced. In addition, gastric cancer clusters with high plasticity have shown a tendency to transition to a high-proliferative phenotype through an autophagy-dependent plasticity program, whereas autophagy inhibitors induced apoptosis in patient-derived organoid (PDO) (Huang et al., 2023). These findings provide insights into the developmental trajectories of cancer and immune cells that underlie GCPM. GIT organoids can be used to recapitulate the entire process of gastric cancer occurrence, development, and metastasis, making them excellent models for gastric cancer research.

In intestinal tumors, Notch signaling plays an important role in regulating tumor progression and metastasis, and it is associated with poor prognosis of CRC intestinal epithelial–mesenchymal transition (EMT). Using intestinal organoids to understand how the Notch pathway regulates epithelial cell regeneration and differentiation is essential for studying intestinal stem cell homeostasis and pathogenesis in intestinal tumors. Fujii et al. (2016) found that Notch signaling activation in organoids alone was not sufficient to induce CRC development. Heuberger et al. (2021) demonstrated that when Notch signaling activation was accompanied by inhibition of p53, it promotes EMT and subsequent tumorigenesis (Chanrion et al., 2014). These studies suggest that GIT tract-like organoids accurately represent physiological signaling during intestinal tumorigenesis, making them valuable tools in studies of intestinal tumor pathogenesis.

### 3.3 GIT organoids for *Helicobacter pylori* studies

*H. pylori* is a major cause of gastric diseases and tumors. The attachment, colonization, and cytotoxin-associated gene A (*CagA*) transport that characterize *H. pylori* infection have been reproduced in organoids. Recognition and attachment of *H. pylori* to target cells is important for its infection and cellular reprogramming. Confocal live cell microscopy has been used to visualize *H. pylori* attachment to infected gastric organoids from normal human mucosa. Based on this, Aguilar et al. used organoids to study *H. pylori* attachment characteristics and found that *H. pylori* preferentially adhered to highly differentiated depressed cells marked by high levels of Gastrokine 1, Gastrokine 2, and the prostate stem cell antigen. Further, this study also showed that attachment was not associated with the expression of Mucin-5AC or the prostate stem cell antigen, but rather depended on TlpB-dependent chemotaxis of the bacterium in response to ureides released by the host cell (Aguilar et al., 2022).

Using organoid models, Sigal et al. (2015) found that *H. pylori* colonizes and manipulates progenitor and stem cell compartments, altering metabolic dynamics and glandular proliferation. This finding has important implications for GIT stem cell biology and *H. pylori*-induced gastric pathology. Furthermore, infection with *H. pylori* strains infused with *CagA* is a major risk factor for death from gastric cancer (Zhang et al., 2022). This strain causes gastric epithelial cell transformation by promoting EMT, which disrupts junctions and enhances the motility and invasiveness of infected cells. This was confirmed in monolayers of cells derived from human organoids, suggesting that *H. pylori* is generally able to transfer *CagA* into organoid cells.

### 3.4 GIT organoids can mimic viral infections

GIT organoids allow patient-derived viruses to exist and replicate efficiently with high accuracy in their disease modeling (Belair et al., 2020; Yin et al., 2015), thereby providing a powerful model system for studying virus-host interactions. These GIT organoids have been used in the study of viral infections capable of causing GIT reactions, including diarrhea [e.g., rotaviruses, Human Norovirus (HuNoV), and SARS-CoV-2] due to their location and functional specificity.

Rotaviruses primarily infect the ileum and jejunum of the host and are capable of destroying enterocytes and impairing intestinal absorption. While we know that rotavirus non-structural protein 4 (NSP4) stimulates intestinal secretion and activates the enteric nervous system, thereby inducing diarrhea, little else is known about the mechanisms behind its pathology. The development of *in vitro* models of the GIT tract will likely be crucial for the study of rotavirus pathogenesis. Finkbeiner et al. used HIOs for the first time for *in vitro* culture of rotaviruses and observed efficient replication of the viruses by immunofluorescence microscopy (Finkbeiner et al., 2012). These data suggest that intestinal organoids are a suitable model for to study rotaviruses.

HuNoV is also an important pathogen in acute gastroenteritis. The development of the HIO culture system provides a new model for further study of HuNoV infection, signaling and pathogenesis. Ettayebi et al. (2016) simulated HuNoV infection in an intestinal organoid model and using immunofluorescence analysis and electron microscopy they confirmed the replication process of this virus in intestinal organoids. Importantly, their data showed that the virus proliferated in the model to produce intact virus particles. This study provides an important experimental model for further research into HuNoV pathology.

Nearly half of patients with SARS-CoV-2 experience GIT symptoms such as diarrhea or nausea, and it is thought that SARS-CoV-2 infection elevates proinflammatory factors that lead to intestinal inflammation (Guo et al., 2021). Krüger et al. (2021) detected virus spiking (S) proteins in 10% of the cells of intestinal organoids after 24 h exposure to SARS-CoV-2, which increased to 57% after 48 h, suggesting virus replication and transmission, which was confirmed by the detection of nucleocapsid (N) proteins. These results suggest that SARS-CoV-2 can effectively infect and replicate in intestinal organoids.

## 4 Drug screening for GIT diseases

Traditionally, most therapeutic drug screening and research campaigns for GIT diseases have been conducted using 2D cell systems. However, there are now an increasing number of studies using GIT organoids for drug screening (Table 1). Compared to 2D cellular systems, GIT organoids provide a more complete picture of the GIT tract at the time of disease, thus increasing the accuracy of drug screening and giving the best options for disease prevention and treatment.

**TABLE 1:** Drug screening for disease (Sort by order of appearance in the article).

Disease	Agents	Dosage	Effect to gastrointestinal organoids	References
IBD	Schisandrin C	5–40 µM	Improves intestinal permeability Enhanced epithelial barrier formation	Kim et al. (2022)
UC	High-concentration acetate	100 mM	Protects intestinal barrier Anti-inflammatory	Deleu et al. (2023)
IBD	(Epi) catechin	0.03–3 mM	Damage to the intestinal epithelium	Guo et al. (2023)
IBD	Metformin	1 mM	Anti-inflammatory and improves intestinal permeability	Hahn et al. (2024)
IBD	*Lactobacillus reuteri*	100 µM	Repairing intestinal damage Maintaining intestinal epithelial regeneration and homeostasis	Wu et al. (2020)
UC	KAG-308	3 mg/kg qd	Anti-inflammatory Promotes regeneration of intestinal epithelial cells Enhances mucus production	Nishimura et al. (2019)
CD	EYTA Butyrate	50 µM 10 mM	Suppression of ECM genes associated with stenosis Suppression of collagen content and tissue stiffness	Jurickova et al. (2022)
CD	Spironolactone	0–250 µM	Blocking the organoid fibrosis response	Rodansky et al. (2015)
CD	Prednisolone	10 µM	Preventing barrier dysfunction	Xu et al. (2021)
HP	lapatinib Cherry-CAP	20 μM NA	Decreased the survival of *H. pylori* in infected organoids	Buti et al. (2020)
Rotavirus	IFN-α Ribavirin	1,000 IU/mL 10 μg/mL	Inhibition of rotavirus replication	Yin et al. (2015)
SARS-CoV-2	Remdesivir	0–100 µM	Inhibition of SARS-CoV-2 replication	Krüger et al. (2021)
Rotavirus	Gemcitabine	0–10 µM	Inhibition of rotavirus replication	Chen et al. (2020)
HuNoV	Ephedra herba	12.5–50 µg/mL	Inhibition of HuNoV replication	Hayashi et al. (2023)
HuNoV	Dasabuvir	5–20 µM	Inhibition of HuNoV replication	Hayashi et al. (2021)
HuNoV	Nitazoxanide	0.1–10 µg/mL	Inhibition of HuNoV replication	Dang et al. (2018)
SARS-CoV-2	Imatinib Mycophenolic acid Quinacrine Dihydrochloride	0.01–100 µM	Inhibition of SARS-CoV-2 replication	Han et al. (2021)
CRC	GANT61 DAPT ATO	10 µM	Enhances 5-Fluorouracil’s chemosensitivity Inhibits invasiveness	Citarella et al. (2023)
CRC	Aspirin	0.5–2 mM	Rescue of the wnt-driven cystic organoid phenotype	Dunbar et al. (2021)
CRC	Butyrate	1 mM	Enhance the efficacy of radiotherapy Protecs the normal mucosa	Park et al. (2020)
GC	55 drugs	NA	Inhibition of tumor cell proliferation	Vlachogiannis et al. (2018)
GC	37 drugs	NA	Inhibition of tumor cell proliferation	Yan et al. (2018)
CRC	83 drugs	NA	Inhibition of tumor cell proliferation	Van De Wetering et al. (2015)
CRC	XAV939 Rapamycin	5 µM 10 µM	Reduce abnormal proliferation	Crespo et al. (2017)
CF	ELX-02	0–160 µM	Restore the CFTR function	Crawford et al. (2021)

### 4.1 IBD treatment and prevention

IBD pathology is associated with intestinal tight junction disorders. Treatment options include targeted therapies such as steroids, aminosalicylates, and tumor necrosis factor-alpha (TNF-α) neutralizing antibodies, but many patients are insensitive to these therapies. Therefore, there is an urgent need for more effective treatments and drugs. An increasing number of studies have shown that intestinal epithelial damage organoids can effectively be used to screen for therapeutic drugs for IBD. In terms of using organoids in identifying preventive measures, Guo et al. (2023) found that excessive intake of (epi) catechins potentially damages the intestinal epithelium in mouse inflammatory intestinal organoids, which may increase the risk of intestinal damage. Thus, limiting or avoiding such risk factors could be an effective preventive measure for IBD.

Intestinal organoid modeling has also been used to highlight reduced intestinal epithelial permeability as a pathological marker of IBD, and therefore, a feature that can be addressed in IBD treatment. Inflammatory cytokines contribute to reduced epithelial permeability, and Hahn et al. used an organoid model of intestinal epithelial injury and found that metformin decreased inflammatory cytokine levels and restored intestinal epithelial permeability (Hahn et al., 2024). Intestinal epithelial permeability is also affected by associated proteins, and Kim et al. (2022) used an intestinal organoid assessment to show that Schisandrin C improved abnormal intestinal permeability and also regulated the expression of proteins closely associated with the development of leaky gut symptoms and IBD, as well as inflammation-associated proteins. Other inducible factors also contribute to intestinal inflammation, Zhang et al. showed that silencing melatonin receptor 1 A inhibited melatonin-induced inflammation in intestinal organoids (Xi et al., 2021). The above drugs play a role in the treatment of IBD by decreasing the levels of inflammatory cytokines, modulating inflammation-related proteins, and inhibiting intestinal epithelial damage-inducing factors, making them important candidates for IBD treatment. In addition to reducing the effects of inflammation-related factors on intestinal inflammation, maintaining intestinal epithelial stability is also crucial for IBD treatment. Wu et al. used intestinal organoids simulating IBD barrier damage to show that *Lactobacillus reuteri* effectively maintains intestinal epithelial cell regeneration and homeostasis and repairs intestinal damage after pathological injury (Wu et al., 2020). These data provide important insights for the development of future UC and CD treatments, which are generally directed at epithelial damage and intestinal fibrosis. The recent advances in organoid technology allow for more accurate predictions of drug effects as well as aiding drug discovery and development. Deleu et al. (2023) used organoids derived from patients with UC to show that high-concentration acetate upregulated *HIF1α, MUC2,* and *MKI67*, while also downregulating most proinflammatory cytokines, which had a protective effect on epithelial resistance, barrier gene expression, and inflammatory protein production. Together, this suggests that high concentrations of acetate are effective therapeutic agents for UC. Nishimura et al. evaluated the therapeutic effects of the drug on intestinal epithelial cells using a colonoid organoid model and showed that the study drug KAG-308 inhibited immune responses and promoted the shift of cellular differentiation towards the secretory profile (Nishimura et al., 2019), suggesting that the KAG-308 could also be a candidate for UC therapy. HIOs provide a platform for testing personalized therapies for CD including small molecule drugs. Jurickova et al. (2022) tested the modulation of mitochondrial and wound healing functions associated with stricturing behavior by small molecules including eicosatetraynoic acid (ETYA) through CD-induced HIOs, and the results showed that in the HIO model ETYA modulated the stenosis-related ECM genes and suppressed collagen content and tissue stiffness, restored mitochondrial function, and promoted wound healing, suggesting a therapeutic effect of ETYA on CD. Rodansky et al. (2015) used HIO as a new model of intestinal fibrosis in CD. The results showed that spironolactone treatment blocked TGFβ-induced fibrosis in HIOs, suggesting that spironolactone can be used to treat CD. Xu et al. (2021) explored the effect of prednisolone on the intestinal-derived organoid epithelial barrier in CD patients and its mechanism, and found that prednisolone played a direct preventive role in cytokine-induced barrier dysfunction by regulating the expression of Claudin-2, E-cadherin, and immunoglobulin-like domain-containing receptor 1. The above drugs were preliminarily screened for their therapeutic effects on UC and CD through GIT organoids, providing additional therapeutic options for clinical treatment.

### 4.2 Drug development for GIT cancer

Chemotherapy and radiotherapy are the main means of CRC treatment, and the use of GIT organoids to simulate the GIT tract conditions in this period provides a powerful model for studying the mechanism of action of traditional drugs in oncology treatment, enhancing chemotherapy sensitivity, improving radiotherapy efficacy, and suppressing the adverse effects of radiotherapy, as well as screening for new therapeutic agents.

Aspirin has been shown to be a chemoprotective agent in the treatment of CRC, but its mechanism of action is not fully understood. Dunbar et al. (2021) used intestinal organoids and found that aspirin restored the Wnt-driven stem cell-like phenotype in HIOs. In addition, 5-Fluorouracil is the main chemotherapeutic agent for CRC but emergence of resistance limits its clinical use. Using GIT tumor organoids, Citarella et al. (2023) found that 5-Fluorouracil promotes mesenchymal cell proliferation and thus invasive phenotypes in *KRAS- and BRAF-*mutant organoids and can be used in combination with Hedgehog/GLI and Notch pathway inhibitors, as well as with GANT61 or arsenic trioxide (ATO) to restore chemosensitivity, suggesting that ATO and GANT61 are promising chemosensitizers in CRC. This study shows the promise of 5-Fluorouracil in lifting clinical therapeutic limitations. These two drugs have been further characterized in organoid-based studies, confirming their roles as effective agents for chemotherapy in CRC and demonstrating the value of GIT organoids as research models for drug improvement.

Radiotherapy is the other mainstay of treatment for GIT tumors, and the use of GIT organoids to improve the efficacy of radiotherapy and reduce radiotherapy-related injury is critical in the treatment of GIT tumors. Park et al. (2020) used organoids derived from patients with CRC to assess their response to radiotherapy, and found that butyrate does not increase radiation-induced cell death and improves regeneration of normal organoids and tissues after radiation. This study suggests that butyrate improves the efficacy of radiotherapy while protecting normal mucosa, a potential strategy to minimize radiotherapy-related toxicity.

In addition to small-scale drug screening for GIT tumors, GIT organoids have also performed well in large-scale drug screening for GIT tumors. Vlachogiannis et al. (2018) studied treatment responses of metastatic GIT cancers using PDOs and screened 55 drugs in these PDOs. They found that PDOs predicted responses to targeted or chemotherapeutic drugs with a sensitivity of 100% and a specificity of response was 93%. These data suggest that PDOs can be used in functional genomics to model tumors and conduct clinical trials. Further, Yan et al. (2018) screened 37 drugs in gastric tumor organoids and found that drugs such as pabukasin (Napabucasin), abemaciclib, an ataxia telangiectasia, and Rad3-related (ATR) kinase inhibitor (VE-822) could be candidates for gastric cancer treatment. van de Wetering et al. (2015) screened 83 drugs using CRC organoids and confirmed the link between drug resistance and genetic mutations. While drugs for GIT tumor therapies are being screened on a large scale for adenomatous polyposis, drug screening for adenomatous polyposis is also underway. Crespo et al. used colonic organoids (COs) as a platform for drug testing and showed that compounds XAV939 and rapamycin reduced proliferation of familial adenomatous polyposis colonic organoids (FAP-Cos). This study also identified a ribosome-binding antibiotic that effectively targeted aberrant WNT activity and specifically restored normal proliferation in *APC*-mutant familial adenomatous polyposis COs (Crespo et al., 2017). This study provides additional lead drug molecule candidates for the treatment of adenomatous polyposis.

### 4.3 *H. pylori* treatment

*Helicobacter pylori* is a major risk factor for gastric cancer, and through GIT organoid studies Buti et al. (2020) found that apoptosis-stimulating protein of p53 2 (ASPP2), a tumor suppressor and important target of *CagA*, contributes to the survival of *CagA*-positive *H. pylori* in the lumen of infected gastric organoid tissues and that it is a key protein in disrupting cell polarity. Studies have shown that inhibiting CagA-positive *H. pylori* ASPP2 signaling with inhibitors of the epidermal growth factor receptor signaling pathway or specific peptides, prevents loss of cell polarity and reduces *H. pylori* survival in infected organoids. These findings suggest that maintaining the host cell polarity barrier reduces the deleterious consequences of *H. pylori* infection, thereby presenting a novel potential strategy for treating *H. pylori* infection.

### 4.4 Drug-mediated inhibition of viral replication

Viral infections often cause acute GIT reactions that can be severe and life-threatening. Rotavirus infection is usually acute and self-limiting, and it can cause chronic infections and serious illness in immunocompromised patients. Gemcitabine is a widely used anticancer drug. Chen et al. (2020) used HIOs to show that gemcitabine is an effective inhibitor of rotaviral infection, which is also beneficial for patients with cancer infected with this virus. In addition, Yin et al. (2015) found that interferon-alpha and ribavirin inhibit rotavirus replication through intestinal organoid studies. These studies suggest that intestinal organoids can be used to evaluate and screen for antiviral drugs.

HuNoV is a major cause of acute gastroenteritis and foodborne illness. GIT organoids can be used to screen for effective drugs to inhibit this virus. Hayashi et al. (2023) screened components of a Japanese–Chinese herbal medicine using stem cell-derived HIOs and found that ephedra herb considerably inhibits HuNoV infection among 22 herbs. Hayashi et al. (2021) used a human intestinal enteroids culture to screen a library of antiviral compounds using this system and successfully identified dasabuvir as a novel anti-HuNoV inhibitor. Using GIT organoids, other teams have found that thiazoles effectively inhibit HuNoV. Dang et al. (2018) discovered that thiazoles inhibit HuNoV replication by inducing the antiviral effector, Interferon regulatory factor-1.

SARS-CoV-2 still poses a serious risk to global health, with up to 50% of patients experiencing GIT symptoms such as diarrhea or nausea. Potent drugs to inhibit the replication of this virus are a key global public health concern. HIOs derived from pluripotent stem cells (PSC-HIOs) as well as colonic organoids (PSC-COs) are important tools for the identification of potent agents against SARS-CoV-2. Krüger et al. (2021) found that rameltegravir effectively inhibits SARS-CoV-2 infection and restores PSC-HIO morphology. Han et al. (2021) used hPSC-COs to perform a high-throughput screening of FDA-approved drugs and identified SARS-CoV-2 inhibitors including imatinib, mycophenolic acid, and quinacrine hydrochloride. Taken together, GIT organoids are clearly important drug screening tools for antiviral drugs, particularly for GIT indications.

### 4.5 Pharmacokinetic and drug toxicology studies

GIT organoids have been cultured to serve as *in vitro* models for exploring drug metabolism, and drug GIT toxicity studies (Lu et al., 2017; Park et al., 2019). Yamada et al. investigated the effects of a novel synthetic lithocholic acid derivative, Dcha-20, with vitamin D activity, on the expression of pharmacokinetic genes in HIOs, and found that Dcha-20 promotes the activity of the intrinsic defense system of intestinal epithelial cells (Yamada et al., 2022). In disease treatment, the decreased activity of the intestinal epithelial defense system makes it more difficult for the body to resist drug-induced GIT toxicity and symptoms such as vomiting and diarrhea. Therefore, research into *in vitro* models of GIT tract toxicity and prediction of drug GIT toxicity is necessary. Belair et al. (2020) used human GIT mesenchymal stromal tumor organoids to show that the model reproduced clinical drug-associated diarrhea with an accuracy of 90%, making it a suitable *in vitro* model for addressing the drug GIT toxicity during preclinical development. The molecular mechanisms of drug-induced GIT toxicity are increasingly being elucidated using GIT organoid models. This is the case for drugs such as gefitinib and Adriamycin (Rodrigues et al., 2022b; Rodrigues et al., 2022a). Further, Lu et al. (2017) used crypt organoid studies to demonstrate that the severe enterotoxicity of the anticancer precursor drug camptothecin-11 originated from the *UGT1A1*-dependent insufficient glucuronidation of its active metabolite, SN-38. Using a mouse intestinal organoid (MIO) model Wang et al. investigated toxicity molecular mechanisms behind the marine toxins, okadaic acid, and conotoxin (CgTx), and found that OA reduced cellular metabolism and energy production by affecting MIO cell transcription, ultimately leading to cell death. In contrast, CgTx upregulates intracellular hormone metabolism pathways by affecting the nuclear receptor pathway of MIO, leading to cell death and high energy production (Wang et al., 2022). An *in vitro* toxicological study of sunset yellow (SY) using an intestinal organoid model by Kong et al. (2021) found that SY disrupts homeostasis in intestinal epithelial cells by producing high levels of the endoplasmic reticulum stress and oxidative stress, and that long-term sustained consumption of SY may increase the risk of intestinal inflammation. Takahashi et al. (2023) developed a lower-cost intestinal organoid and found that YC-1 [3-(5′-hydroxymethyl-2′-furyl)-1-benzyl indazole] induces apoptosis through the mitogen-activated protein kinase/extracellular signal-regulated kinase pathway. It is the high degree of restoration of the physiological structure and function of the GIT tract that has led to the use of GIT organoids in a wide range of toxicological studies.

## 5 GIT organoids in microbiology research and clinical trials

GIT organoids are not only a platform for screening drugs for GIT diseases, but also play a role in immunomodulation, gut microbiota research, and the treatment of post-radiation intestinal epithelial damage, cystic fibrosis (CF), and diabetes. Importantly, GIT organoids also have an immunomodulatory role, Zhang et al. (2021) isolated extracellular vesicles using mouse and human GIT organoids and found that EVs play a crucial role in maintaining homeostasis in the host. The gut microbiota plays an important role in the formation of the intestinal immune system, and it has been found that in HIOs, 13-hydroxy-cis-6,cis-9-octadecadienoic acid (γHYD), and 13-oxo-cis-6,cis-9-octadecadienoic acid (γKetoD) produced by *Lactobacillus intestinalis*, which are naturally occurring peroxisome proliferator-activated receptor delta ligands in the intestinal tract, are able to promote the β-oxidation of fatty acids and to reduce the accumulation of intracellular triglycerides, in order to improve lipid metabolism of the human intestinal tract (Noguchi et al., 2022).

GIT organoids are widely used in clinical trials and have been used as an important tool in regenerative medicine and as a medication guide for clinical treatment. Radiation therapy is commonly used for GIT tumors, and high-dose radiation exposure induces GIT stem cell death, leading to intestinal mucosal denudation and GIT syndrome death. In recent years, drugs to attenuate radiotherapy injury have been screened through intestinal organoid studies, and experiments using intestinal organoid transplantation cells to treat radiotherapy injury have been successful. Wang et al. (2020) used intestinal injury organoids and found that present arachidonic acid activated radiation-resistant Musashi-1+ cells promote intestinal epithelial repair. Fu et al. (2021) used intestinal organoids and found that knockdown or drug inhibition of sirtuin1 increased p53 acetylation and led to p53 stabilization, which considerably improved the survival of irradiated intestinal epithelial cells, suggesting that sirtuin1 inhibitors are an effective clinical countermeasure to attenuate intestinal damage caused by radiation exposure. Moussa et al. (2020) found that *in vitro* expanded epithelial cells transplanted from mouse colonoid organoids implanted, proliferated, and differentiated in irradiated mucosa and reduced ulcer size. This study demonstrates the potential of organoids to limit the effects of late radiation on the colon and opens the prospect of a combined strategy to improve their expansion capacity and therapeutic efficacy.

In a therapeutic study of CF, researchers used PDOs derived from patients with the *G542X* genotype and found that ELX-02 targeting of the *G542X* cystic fibrosis transmembrane conductance regulator (CFTR) nonsense allele restored CFTR function in HIOs (Crawford et al., 2021), supporting the clinical evaluation of ELX-02 as a through-putting agent for the treatment of CF caused by mutations in the *G542X* allele. Organoids are not only used to investigate CF therapeutic agents but also to guide the treatment for patients with clinical CF. Forskolin-induced swelling of patient organoids was used to measure patient-specific CFTR function and CFTR modulator response and has been used to clinically guide the treatment of a patient with a rare genotype of CFTR mutation (Aalbers et al., 2022).

GIT organoids can also be used in diabetes treatment research, supporting the discovery of diabetes therapeutic targets and the restoration of glucose homeostasis *in vivo*. Filippello et al. (2022) used MIOs that mimic lipotoxicity to find that lipotoxicity affects the differentiation of specific intestinal cell types in the intestinal tract, and also identified new targets related to the molecular mechanisms affected by lipotoxicity that may be important for the treatment of obesity and diabetes. Huang et al. (2023) cultured islet organoids differentiated from human gastric stem cells containing gastric insulin-secreting cells with similar molecular characteristics and function to β-cells. The organoids were found to acquire glucose-stimulated insulin secretion within 10 days and to restore glucose homeostasis in diabetic mice within 100 days post-transplantation, providing a potentially promising new approach to diabetes treatment.

## 6 Summary and outlook

In this review we discuss the application of GIT organoid technology in disease research and drug screening. GIT tract organoids maintain the genetic properties, physiological structure, and function of the GIT tract, have the ability to accurately model GIT diseases, and have great potential for aiding our understanding of disease pathology and for developing new treatments for GIT diseases. Due to their miniaturization and ability to mimic the physiological structure and function of the GIT tract, coupled with their amenability to high-throughput screening and the emergence of conditioned media and tissue-derived organoids that have greatly reduced the time and cost of cultivation, GIT organoids are invaluable tools for predicting preclinical drug toxicity and screening for clinical therapeutic agents.

Although GIT organoids offer advantages for basic research and clinical applications, they still have limitations, including differences in drug exposure compared to physiological conditions, the absence of an immune system, insufficient precision in responses to modulators, difficulties in replicating the gastrointestinal tumor microenvironment, and ethical issues associated with tissue collection. First, drug exposure in organoids differs from *in vivo* GIT administration; typically, drug exposure occurs on the basolateral side. Using microfluidic devices or more precise drug delivery systems in experiments to ensure that drugs are exposed accurately to the organoids from the apical side can address this issue. Second, organoid models contain cell types that are limited to intestinal epithelial cells, restricting studies on immune responses and the effects of drugs on immune cells within organoids. Technologies for incorporating immune system components into organoid systems are still underdeveloped, require further research. Furthermore, GIT organoid responses to modulators exhibit limited precision, especially in small differences, which requires us to enhance the precision of experimental design, such as establishing uniform standards for organoid culture and experimental procedures, in order to reduce variability between experiments. Additionally, GIT organoids cannot fully replicate the GIT tumor microenvironment or achieve purification of GIT tumor organoids. To address this challenge, we can reconstruct the extracellular matrix using biomaterials and specific extracellular matrix components, regulate key biochemical factors, and perform gene editing and epigenetic modifications. These actions are intended to maximize the simulation of the extracellular matrix of tumor cells and genetic alterations in cancer. At the same time, by employing methods such as flow cytometry, immunomagnetic bead sorting, fluorescent protein labeling, and microfluidic technology, we can achieve the spatial separation and purification of tumor cells, enhancing the purity of organoid tumor cells. Finally, generating healthy organoids from the same individual for tissue or donor specificity studies poses ethical challenges due to the need for donor to undergo nonessential surgical procedures. Therefore, ethical review and informed consent are very necessary.

Despite the limitations of GIT organoids, a growing number of studies have confirmed their potential for personalized treatment of GIT diseases. Studies combining microvascularized intestinal organoids and GIT organoids with gene editing technologies have provided more accurate models for GIT disease research. Large-scale cancer organoid biobanks have already been established, and to further improve precision medicine for GIT diseases, GIT organoid biobanks should be established to allow for faster preclinical studies of drugs and provide personalized medication guidance for patients with GIT diseases.

## Abbreviations

ASCs, adult stem cells; *CagA*, cytotoxin-associated gene A; CD, crohn’s disease; CF, cystic fibrosis; CFTR, cystic fibrosis transmembrane conductance regulato; CRC, colorectal cancer; EMT, epithelial-mesenchymal transition; EYTA, eicosatetraynoic acid; FAP-Cos, familial adenomatous polyposis - colonic rganoids; GIT, gastrointestinal tract; HIE, human intestinal enteroids; HIOs, human intestinal organoids; *H. plori, Helicobacter pylori*; PSC-Cos, colonic organoids derived from pluripotent stem cells; HuNov, Human Norovirus; IBD, inflammatory bowel disease; IFN-α, interferon-alpha; MIO, mouse intestinal organoid; PDOs, patient-derived organoids; PSCs, pluripotent stem cells; PSC-HIO, human intestinal organoids derived from pluripotent stem cells; SNPs, single-nucleotide polymorphisms; UC, ulcerative colitis.
